# Association Between Diabetic Retinopathy and Cognitive Impairment: A Systematic Review and Meta-Analysis

**DOI:** 10.3389/fnagi.2021.692911

**Published:** 2021-06-30

**Authors:** Dihe Cheng, Xue Zhao, Shuo Yang, Guixia Wang, Guang Ning

**Affiliations:** ^1^Department of Endocrinology and Metabolism, The First Hospital of Jilin University, Changchun, China; ^2^Key Laboratory for Endocrine and Metabolic Diseases of Ministry of Health of China, Shanghai National Clinical Research Center for Endocrine and Metabolic Diseases, Shanghai Institute for Endocrine and Metabolic Diseases, Ruijin Hospital, Shanghai Jiaotong University School of Medicine, Shanghai, China

**Keywords:** diabetes mellitus, diabetic retinopathy, cognitive impairment, mild cognitive impairment, dementia

## Abstract

Diabetic retinopathy (DR) is one of the most common microvascular complications associated with diabetes mellitus. However, its correlation with another diabetes-related disorder, cognitive impairment, has not been well studied. This systematic review and meta-analysis aimed to explore the association between DR and cognitive impairment. MEDLINE (PubMed), the Cochrane Library, and EMBASE databases were searched for observational studies that reported an association between DR and cognitive impairment. Data from selected studies were extracted, and a meta-analysis was conducted using fixed-effects modeling. Fifteen observational studies were included in the systematic review, and 10 studies were included in the meta-analysis. The odds ratio of the association between DR and cognitive impairment was 2.24 (95% confidence interval [CI], 1.89–2.66; *I*^2^ = 0.8%). The hazard ratio of the association between DR and cognitive impairment was significant in four studies, ranging from 1.09–1.32. Minimal or mild DR was not significantly associated with cognitive impairment (odds ratio [OR], 2.04; 95% CI, 0.87–4.77). However, the association between proliferative DR and cognitive impairment (OR, 3.57; 95% CI, 1.79–7.12; *I*^2^ = 16.6%) was not stronger than the association between moderate or worse DR and cognitive impairment (OR, 4.26; 95% CI, 2.01–9.07; *I*^2^ = 0.0%). DR is associated with cognitive impairment, and screening for DR will be helpful for the early identification of individuals with cognitive impairment. Further studies are needed to confirm the association between proliferative DR and cognitive impairment.

## Introduction

Diabetes mellitus is a group of metabolic diseases characterized by hyperglycemia caused by defective secretion of insulin, the impaired biological action of insulin, or both. In 2015, 415 million people were estimated to have diabetes, with a projected increase to 642 million by 2040 (Chatterjee et al., 2017). The overall prevalence of diabetic retinopathy (DR) is 35% among patients with diabetes worldwide (Hammes, 2018). As one of the most common microvascular complications of diabetes mellitus, screening for DR has been widely performed in clinical practice.

Cognition is a process in which the human brain receives information from the outside world, processes it, and transforms it into internal psychological activities to acquire or apply knowledge. It includes memory, language, visual space, execution, computation, understanding, and judgment. Cognitive impairment mainly includes mild cognitive impairment and dementia. Mild cognitive impairment (MCI) is defined as acquired cognitive complaints with objective abnormal test results in one or more domains on formal cognitive testing, and dementia is defined as the most severe stage of cognitive dysfunction, with objective impairment of multiple cognitive domains, by definition affecting activities of daily life (Biessels and Whitmer, 2020). Although the lack of standardized diagnostic criteria and differences in the characteristics of different study samples lead to significant uncertainties in these estimates, the prevalence of MCI is approximately 10–20% (Langa and Levine, 2014), and the incidence of dementia is ~7% in people over 65 years of age (Prince et al., 2013).

Both cognitive impairment and diabetes mellitus are closely associated with aging. Although cognitive impairment is not unique to diabetes, diabetes-related cognitive impairment is now recognized as a complication of diabetes. The risk of incident MCI (up to 60%) and dementia (50–100%) is higher in patients with type 2 diabetes than in those without (Srikanth et al., 2020). In one retrospective study, the risk of incident dementia in hospital-admitted patients with type 1 diabetes was 1.65 times higher than that in people without diabetes (Smolina et al., 2015). Although the risk of developing cognitive impairment in diabetes has received much attention, clinical guidelines have recently begun to emphasize its importance.

The prediction and identification of cognitive impairment in individuals with diabetes will be helpful in early intervention. Furthermore, since the eyes are the “window” to the brain, damage to the retina may be a sign of neurodegenerative diseases of the brain (Simó et al., 2018). Therefore, if DR is associated with cognitive impairment, it will be beneficial for predicting and preventing diabetes-related cognitive impairment and further emphasizes the importance of DR screening.

However, the relationship between DR and cognitive impairment has not been fully studied, and the findings are ambiguous. To date, one systematic review of DR and cognitive impairment in patients with type 2 diabetes has been published (Crosby-Nwaobi et al., 2012), but only three studies were included in the review, and there was a lack of population-based cohort studies. Therefore, the present systematic review and meta-analysis aimed to explore the association between DR and cognitive impairment as well as the association between the grades of DR and cognitive impairment.

## Methods

### Literature Search Strategy

Relevant studies were identified by systematically searching MEDLINE (PubMed), the Cochrane Library, and EMBASE databases from inception to November 6, 2020 (date last searched), using a combination of Medical Subject Heading terms with related free-text terms (“diabetic retinopathy,” “cognitive dysfunction,” “dementia,” “Alzheimer disease”). Additional articles were identified via manual search of the reference lists of relevant articles and previous review articles.

During this process, two independent investigators (Cheng and Zhao) completed this work to reduce selection bias. If there were disputes, a third investigator (Wang) resolved the disagreements.

### Inclusion and Exclusion Criteria

Articles were included if they fulfilled the following criteria: 1) were cohort study, cross-sectional study, or case-control study design; 2) had DR as the exposure of interest; 3) included people without diabetic retinopathy as the control group; 4) had cognitive impairment including dementia as an outcome of interest; and 5) odds ratios (ORs) or hazard ratios (HRs) and 95% confidence intervals (CIs) were reported or could be calculated. The studies were limited to those conducted in the human population and English. Studies were excluded if categorized as editorials, literature reviews, case reports, and conference abstracts. Included and excluded studies were collected following the Preferred Reporting Items for Systematic Reviews and Meta-Analyses (PRISMA) flow diagram (Moher et al., 2009). The current systematic review and meta-analysis followed the Meta-analysis of Observational Studies in Epidemiology guidelines for meta-analysis of observational studies (Stroup et al., 2000).

### Study Selection and Data Extraction

Eligible studies were assessed for overlap based on authors, study region, study population, sample size, and variable measurements. If there was an overlap of the study groups, articles of better quality were selected for the analysis. In addition, the following information was extracted from each study: authors, year of publication, country, study design, definition of cognitive impairment and DR, participant characteristics, sample size, outcome of interest, adjusted confounders (if possible), and duration of follow-up (if possible). During this process, two independent investigators independently screened the titles and abstracts of the identified searches, followed by a full-text review of potentially eligible articles to reduce selection bias.

### Quality Assessment

The Newcastle-Ottawa Scale was used in the current systematic review and meta-analysis to evaluate the quality of cohort studies and case-control studies in terms of study group selection, group comparability, and exposure or outcome of interest (Stang, 2010). The scale uses a star system (with a maximum of nine stars). Studies with stars 0–3 were considered as “low quality,” with stars 4–6 were considered as “moderate quality,” and with stars 7–9 were considered as “high quality.” We also evaluated the quality of cross-sectional studies according to the standards recommended by the Agency for Healthcare Research and Quality (https://www.ncbi.nlm.nih.gov/books/NBK35156/). The methodological quality of the included studies was assessed using an 11-item checklist. An item would be scored “0” if it was answered “NO” or “UNCLEAR;” if it were answered “YES,” then the item would be scored “1.” Thus, studies with scores 0–3 were considered of “low quality,” with scores 4–7 were considered of “moderate quality,” and with scores 8–11 were considered of “high quality.” Two authors (Cheng and Yang) independently assessed the risk of bias.

### Data Synthesis and Analysis

Summary estimates and corresponding 95% CIs for the outcome of the relationship between DR and the risk of cognitive impairment were pooled, if possible. In the case of studies reporting ORs or HRs with various degrees of adjustment, we always used fully adjusted estimates and their 95% CIs. For studies that did not estimate ORs of the relationship between DR and the risk of cognitive impairment, we calculated the unadjusted ORs and 95% CIs using a two-by-two table. A fixed-effects model was used to pool the ORs across the selected studies. Cochran's *Q*-test and I^2^ statistics were used to quantify heterogeneity, with values of *I*^2^ > 50% representing medium heterogeneity (Lijmer et al., 2002; Li et al., 2015). We also performed a subgroup analysis to identify potential effect modifiers. If a study included multiple grades of DR, each result was analyzed separately in the subgroup analysis. Subgroup analyses by study type, duration of follow-up, and whether confounding factors had been adjusted (studies that provided unadjusted ORs and 95% CIs were grouped with those in which we calculated ORs using a two-by-two table) were also performed. Given the expected heterogeneity of the eligible studies, a sensitivity analysis was performed. We used the funnel plot and the Egger test to evaluate publication bias. A *P*-value of less than 0.05 was considered statistically significant. All analyses were conducted with STATA 15.1. Due to the high heterogeneity, a quantitative meta-analysis could not be performed to pool HRs across the selected studies.

## Results

### Literature Search

**Figure 1:**
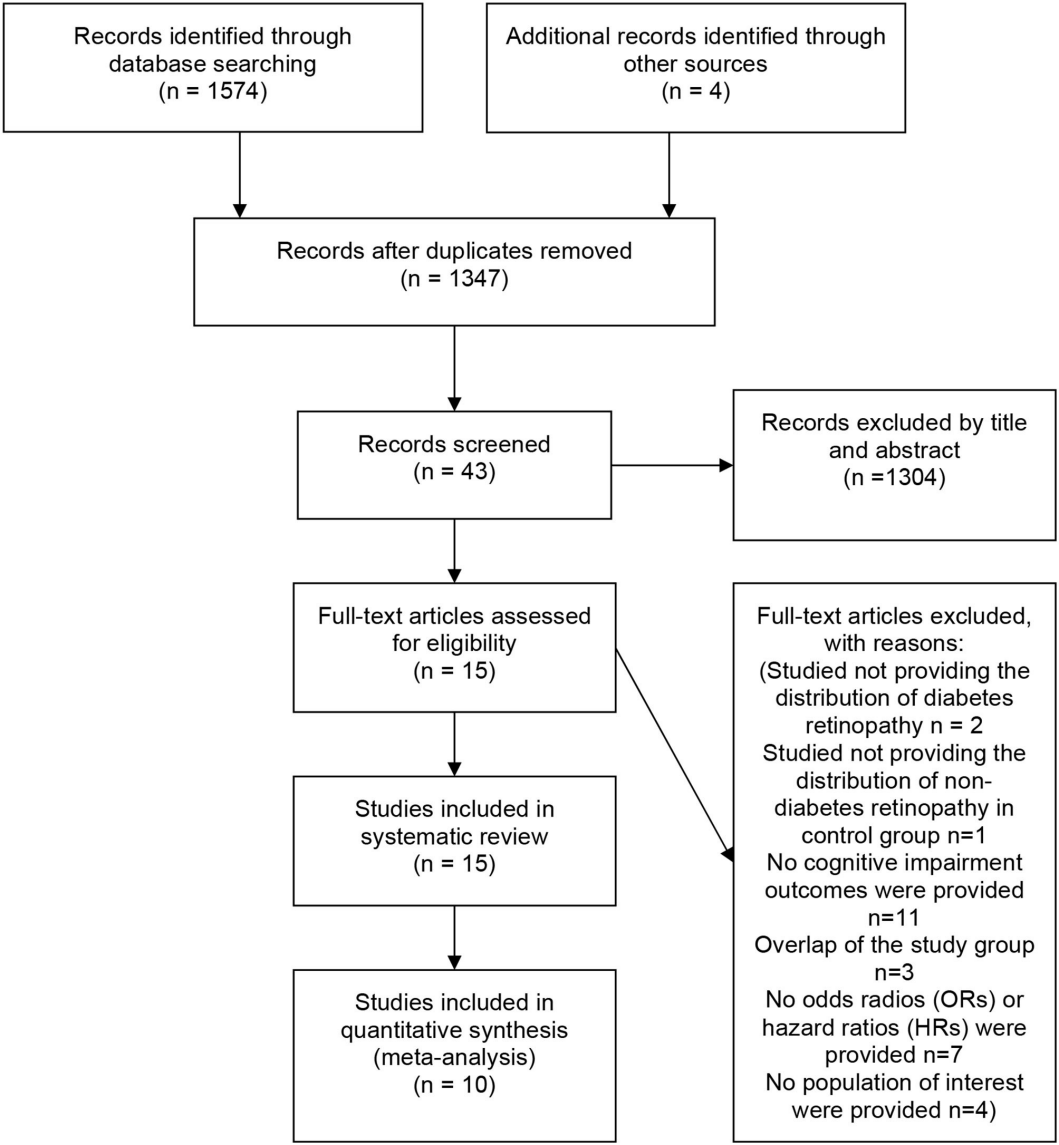
PRISMA flow diagram of this systematic review and meta-analysis.

The initial literature search yielded 1574 articles. Four additional articles were identified via manual search. Among all articles, 231 were duplicates. After screening the abstracts and titles, 43 articles remained. After a full-text review, 27 studies were excluded for the reasons specified in the PRISMA diagram. Fifteen observational studies were included in the systematic review, and 10 studies were included in the meta-analysis (Figure 1).

### Study Characteristics

As summarized in Table 1, among the eligible studies reporting ORs and 95% CIs, four studies had a cross-sectional study design (Baker et al., 2007; Umegaki et al., 2008; Ogurel et al., 2015; Xia et al., 2020), two studies had a case-control study design (Roberts et al., 2008; Gorska-Ciebiada et al., 2015), and four studies had a cohort design (Kadoi et al., 2005; Bruce et al., 2014; Nunley et al., 2015; Gupta et al., 2019). Overall, there were 4769 adult participants among the 10 studies included in the meta-analysis. A total of five studies were carried out in Asia (China, Singapore, Turkey, and Japan) (Kadoi et al., 2005; Umegaki et al., 2008; Ogurel et al., 2015; Gupta et al., 2019; Xia et al., 2020), one study was carried out in Europe (Gorska-Ciebiada et al., 2015), two studies were carried out in North America (Roberts et al., 2008; Nunley et al., 2015), and two studies were carried out in Oceania (Baker et al., 2007; Bruce et al., 2014). Most of these studies were conducted on older subjects. Among the included studies, three studies used the Early Treatment of Diabetic Retinopathy Study (ETDRS) or modified ETDRS criteria to define DR (Kadoi et al., 2005; Ogurel et al., 2015; Gupta et al., 2019), four studies used other methods to define DR (Baker et al., 2007; Umegaki et al., 2008; Bruce et al., 2014; Nunley et al., 2015), and three studies did not mention how to define DR (Roberts et al., 2008; Gorska-Ciebiada et al., 2015; Xia et al., 2020). The control groups of the nine studies included the diabetic population (Kadoi et al., 2005; Baker et al., 2007; Umegaki et al., 2008; Bruce et al., 2014; Gorska-Ciebiada et al., 2015; Nunley et al., 2015; Ogurel et al., 2015; Gupta et al., 2019; Xia et al., 2020), while the control group of one study consisted of a non-diabetic population (Roberts et al., 2008).

**Table 1:** Characteristics of the study providing odds ratios.

**References**	**Country**	**Definition of diabetic retinopathy**	**Definition of cognitive impairment**	**Participant characteristics**	**Enrolled sample number**	**Study design**	**Follow-up**	**OR (95%)**	**Adjusted confounders**
Xia et al. (2020)	China	Not mentioned	CDR score was bounded by 1.0/0.5 for dementia, 0.5/0 for MCI. MMSE score was bounded by 23/24 for dementia, and MOCA score by 25/26 for MCI. For the participants who had 12 years of education or fewer, a point was added to his/ her total MOCA score.	Hospital patients: T2DM subjects aged 45–74 years old	DR: 146 Control: 151	Cross-sectional	**/**	**Dementia:** DR: 2.197 (1.035–4.664)	Age, sex and education level.
Gupta et al. (2019)	Singapore	Modified airlie house classification system: none (early treatment of diabetic retinopathy study level 10), minimal/mild (level 20–35) and moderate or worse DR (level 43–90) using data from the better eye.	Validated AMT: scores of ≤6 and ≤8 for those with 0–6 and >6 years of formal education.	SEED-1 study: participants with diabetes who were ≥60 years	DR :199 Control: 483	Cohort	6 years	**Cognitive impairment:** DR: 2.32 (1.07–5.03) Minimal or mild DR: 2.04 (0.87–4.76) Moderate or worse DR: 3.41 (1.06 −11.00)	Age, gender, race, education, income, spherical equivalent, HbA1c, diabetes duration, hypertension, CVD and presence of eye conditions (cataract, age-related macular degeneration, glaucoma and undercorrected refractive error in the better eye), better eye presenting visual acuity.
Ogurel et al. (2015)	Turkey	The criteria of the early treatment diabetic retinopathy study.	The cut off score <21 on the MoCA.	Patients with diabetes	DR: 90 Control: 30	Cross-sectional	**/**	**Cognitive impairment:**DR: 3.5 (1.464-8.365) Moderate or worse DR: 5.0 (1.864-13.409) PDR: 6.5 (1.820-23.213)	No
Nunley et al. (2015)	USA	Proliferative retinopathy was defined as receiving laser therapy for proliferative diabetic retinopathy.	Individual raw test scores ≥1.5 SD worse than published norms (Ardila, 2007; Dominic, 2007; Dore et al., 2007).	EDC study: middle-aged adults with T1DM diagnosed before age 18 years	DR: 46 Control: 51	Cohort	About 27 years	**Cognitive impairment:** PDR: 2.79 (1.23–6.33)	Years of education
Gorska-Ciebiada et al. (2015)	Poland	Not mentioned	MCI was diagnosed based on criteria established by the 2006 European Alzheimer's Disease Consortium.	Hospital patients: Patients aged 65 and over with T2DM	DR: 121 Control: 155	Case-control	**/**	**MCI:** DR: 2.24 (1.7–2.96)	No
Bruce et al. (2014)	Australia	Ophthalmoscopy and/or detailed specialist assessment or retinal photography.	Normal cognition (CDR 0), cognitive impairment but not demented (CDR 0.5), and mild/moderate/severe dementia (CDR 1–3)	FDS study: T2DM patients aged 50 years or more	DR: 30 Control: 290	Cohort	14.7 years	**Cognitive impairment:** DR: 3.11 (1.15–8.38)	No
Umegaki et al. (2008)	Japan	Diabetic retinopathy was classified into two categories: mild (no retinopathy or intraretinal hemorrhages and hard exudates), or serious (soft exudates, intraretinal microvascular abnormalities, venous caliber abnormalities, venous beading, neovascularization of the disc or other areas in the retina, preretinal fibrous tissue proliferation, preretinal or vitreous hemorrhage, and/or retinal detachment).	Having an MMSE score of 23 or less.	J-EDIT study: Japanese people with diabetes aged 65 years or more	DR: 448 Control: 459	Cross-sectional	**/**	**Cognitive impairment:** DR: 1.730 (0.998–2.997)	Age
Roberts et al. (2008)	USA	Not mentioned	MCI was defined according to the following published criteria: cognitive concern by physician, patient, or nurse; impairment in 1 or more of the 4 cognitive domains; essentially normal functional activities; and not demented. A diagnosis of dementia was based on the Diagnostic and Statistical Manual of Mental Disorders, 4th edition criteria.	Olmsted county residents: subjects were found to be free of dementia aged 70 through 89 years	DR: 43 Control: 1558	Case-control	**/**	**MCI:** DR: 2.15 (1.09-4.22)	Age, sex, education, hypertension, stroke or transient ischemic attack, cigarette smoking, coronary artery disease, and body mass index.
Baker et al. (2007)	Australia	The photographs were evaluated according to a standardized protocol into 4 broad categories for: (1) retinopathy signs (microaneurysms, retinal hemorrhages, cotton wool spots, hard exudates, macular edema, intraretinal microvascular abnormalities, venous beading, new vessels at the disc or elsewhere, and vitreous hemorrhage); (2) arteriovenous nicking; (3) focal arteriolar narrowing; and (4) retinal arteriolar and venular caliber.	Definition of dementia correlates very closely to criteria used in the Diagnostic and Statistical Manual of Mental Disorders, 4th edition.	CHS study: diabetes adults 65 years of age and older	Total: 289	Cross-sectional	**/**	**Dementia:** DR: 0.32 (0.07- 1.44)	Not mentioned
Kadoi et al. (2005)	Japan	A modification of the diabetic retinopathy study and the early treatment diabetic retinopathy study grading scale.	Cognitive functioning was assessed with the following tests: (1) Mini-Mental State Examination, (2) Rey Auditory Verbal Learning Test, (3) Trail-Making Test (part A), (4) Trail-Making Test (part B), (5) Digit Span Forward, and (6) Grooved Pegboard. Significant impairment was defined as a decline from preoperative testing of more than 1 SD on more than 20% of test measures (at least 2 of 6).	Hospital patients: patients with T2DM who were scheduled for elective coronary artery bypass grafting	DR: 51 Control: 129	Cohort	7 days and 6 months	**All cognitive impairment:**DR: 2.4 (1.4-2.9) **Short-term cognitive impairment:**DR: 2.0 (1.3-3.0) **Long-term cognitive impairment:**DR: 2.1 (1.2-2.7)	No

*CDR, clinical dementia rating; MCI, mild cognitive impairment; MMSE, Mini-Mental State Examination; MOCA, montreal cognitive assessment; T2DM, type 2 diabetes mellitus; DR, diabetic retinopathy; AMT, abbreviated mental test; HbA1c, glycosylated hemoglobin; CVD, cardiovascular disease; T1DM, type 1 diabetes mellitus; PDR, proliferative diabetic retinopathy*.

As summarized in Table 2, among the eligible studies reporting HRs and 95% CIs, all five studies had a cohort design (Exalto et al., 2014; Rodill et al., 2018; Deal et al., 2019; Lee et al., 2019; Yu et al., 2020), and four of them were carried out in the USA (Exalto et al., 2014; Rodill et al., 2018; Deal et al., 2019; Lee et al., 2019). Two studies used the same database but different populations (Exalto et al., 2014; Rodill et al., 2018). In these five studies, there were 1,957,187 participants (10.2% with DR, *n* = 200,323) aged 40 years or older. The mean duration of follow-up ranged from 5.1 to 16 years. All studies conducted multivariable-adjusted analyses with important confounders, including age and sex. The outcomes were dementia, including Alzheimer's disease and vascular dementia. Due to the high heterogeneity, a quantitative meta-analysis could not be performed to pool HRs across the selected studies.

**Table 2:** Characteristics of the study providing hazard radios.

**References**	**Country**	**Definition of diabetic retinopathy**	**Definition of cognitive impairment**	**Participant characteristics**	**Enrolled sample number**	**Study design**	**Mean follow-up**	**HR (95%)**	**Adjusted confounders**
Yu et al. (2020)	Korea	ICD-10 code H36.0.	Prescribed anti-dementia medications (rivastigmine, galantamine, memantine, or donepezil) along with ICD-10 codes (F00, F01, F02, F03, G30, or G31).	NHIS: 40 years of age or older with diabetes	DR: 195449 Control: 1722253	Cohort	5.1 years	**Any type of dementia:**DR: 1.09 (1.07–1.11) **Alzheimer's disease:**DR: 1.10 (1.07–1.12) **Vascular dementia:**DR: 1.08 (1.03–1.14)	Age, sex, smoking, alcohol intake, exercise, income, plasma glucose concentration, duration of diabetes, BMI, dyslipidemia, hypertension, diabetic retinopathy, CKD, stroke, IHD, depression, number of OHAs, and treatment with insulin.
Deal et al. (2019)	USA	Modified Airlie House classification, as used in the Early Treatment Diabetic Retinopathy Study.	Modified CDR interviews with informants confirming a hospital ICD-9 discharge or death certificate dementia code, or on hospital or death certificate dementia codes alone.	ARIC study: Diabetes aged 50-73 years	DR: 324 Control: 1581	Cohort	16 years	**Dementia:**Moderate/severe retinopathy: 2.14 (1.50–3.04) Mild retinopathy: 1.5(0.9-2.5)	Age (linear and quadratic terms), education, sex, race center interaction, BMI, drinking status, smoking status, diabetes, hypertensive status, CHD, and history of stroke.
Lee et al. (2019)	USA	ICD-9 codes: DR (362.01, 363.02, 362.03, 362.04, 362.05, 362.06).	late-onset clinical Alzheimer's disease as defined by NINCDS-ADRDA criteria. Dementia diagnoses were determined at consensus conferences using the Diagnostic and Statistical Manual of Mental Disorders, 4th Version, criteria.	ACT participants (Kaiser Permanente Washington membership): Adults aged ≥65 who were dementia-free	DR: 248 Control: 3629	Cohort	>8 years	**Alzheimer's disease**:0–5 years: 1.67 (1.01, 2.74) More than 5 years: 1.50 (1.05, 2.15)	Age, sex, education, self-reported white race, any APOE ε4 alleles, and time-dependent smoking status.
Rodill et al. (2018)	USA	ICD-9 diagnostic and CPT-4 procedural codes were used. PDR (ICD-9: 362.02; CPT-4: 67228), macular edema (ICD-9: 362.07, 362.53, 362.83; CPT-4: 67208, 67210) or nonspecific DR (ICD-9: 250.5x, 362.0x).	Dementia were identified using the following ICD-9 diagnostic codes: Alzheimer disease (331.0), vascular dementia (290.4x), and nonspecific dementia (290.0, 290.1x, 290.2x, 290.3, 294.1x, 294.2x, and 294.8).	KPNC database: Members with T1DM, with no prevalent dementia diagnoses, and at least 50 years old	DR: 2294 Control: 1448	Cohort	6.2 years	**Dementia:** DR: 1.12 (0.82, 1.54)	Age, sex and race, baseline glycosylated hemoglobin and comorbidities.
Exalto et al. (2014)	USA	PDR: panretinal photocoagulation to treat proliferative retinopathy (CTP4 code 67228), for diabetic macular edema: focal and grid photocoagulation to treat macular edema (CTP4 codes 67208 67210); or outpatient diagnoses made in ophthalmology: (ICD 9 codes 250.5 + 362.02 for PDR; ICD 9 codes 250.5 + 362.53 or 250.5 + 362.83 for diabetic macular edema).	Dementia was identified using ICD-9-CM diagnosis codes; senile dementia uncomplicated (290.0), Alzheimer disease (331.0), vascular dementia (290.4x), and dementia not otherwise specified (290.1). Using diagnoses made in primary care (ICD 9 codes 290.0, 290.1x) and neurology or memory clinic visits (ICD 9 codes 331.0, 290.1x, 290.2x, 290.3, 290.4x).	KPNC database: Patients aged ≥60 years with T2DM	DR: 2008 Control: 27953	Cohort	6.6 years	**Dementia:** DR: 1.32 (1.17, 1.49)	Age (as time scale), gender, race and education, medical utilization, diabetes mellitus composite, vascular composite, BMI and smoking status.

*ICD, international classification of diseases; DR, diabetic retinopathy; BMI, body mass index; CKD, chronic kidney disease; IHD, schemic heart diseases; OHAs, oral hypoglycemic agents; CDR, Clinical Dementia Rating; CHD, coronary heart disease; NINCDS-ADRDA, national institute of neurological and communicative disorders and stroke–alzheimer's disease and related disorders association; APOE, apolipoprotein E; CPT-4, current procedural terminology, 4th edition; PDR, proliferative diabetic retinopathy; T1DM, type 1 diabetes mellitus; T2DM, type 1 diabetes mellitus*.

Of the 15 included studies, six were of fair quality (Roberts et al., 2008; Exalto et al., 2014; Rodill et al., 2018; Deal et al., 2019; Gupta et al., 2019; Lee et al., 2019), while others were of moderate quality (Kadoi et al., 2005; Baker et al., 2007; Umegaki et al., 2008; Bruce et al., 2014; Gorska-Ciebiada et al., 2015; Nunley et al., 2015; Ogurel et al., 2015; Xia et al., 2020; Yu et al., 2020) (Supplementary Tables 1, 2).

### DR and Cognitive Impairment

**Figure 2:**
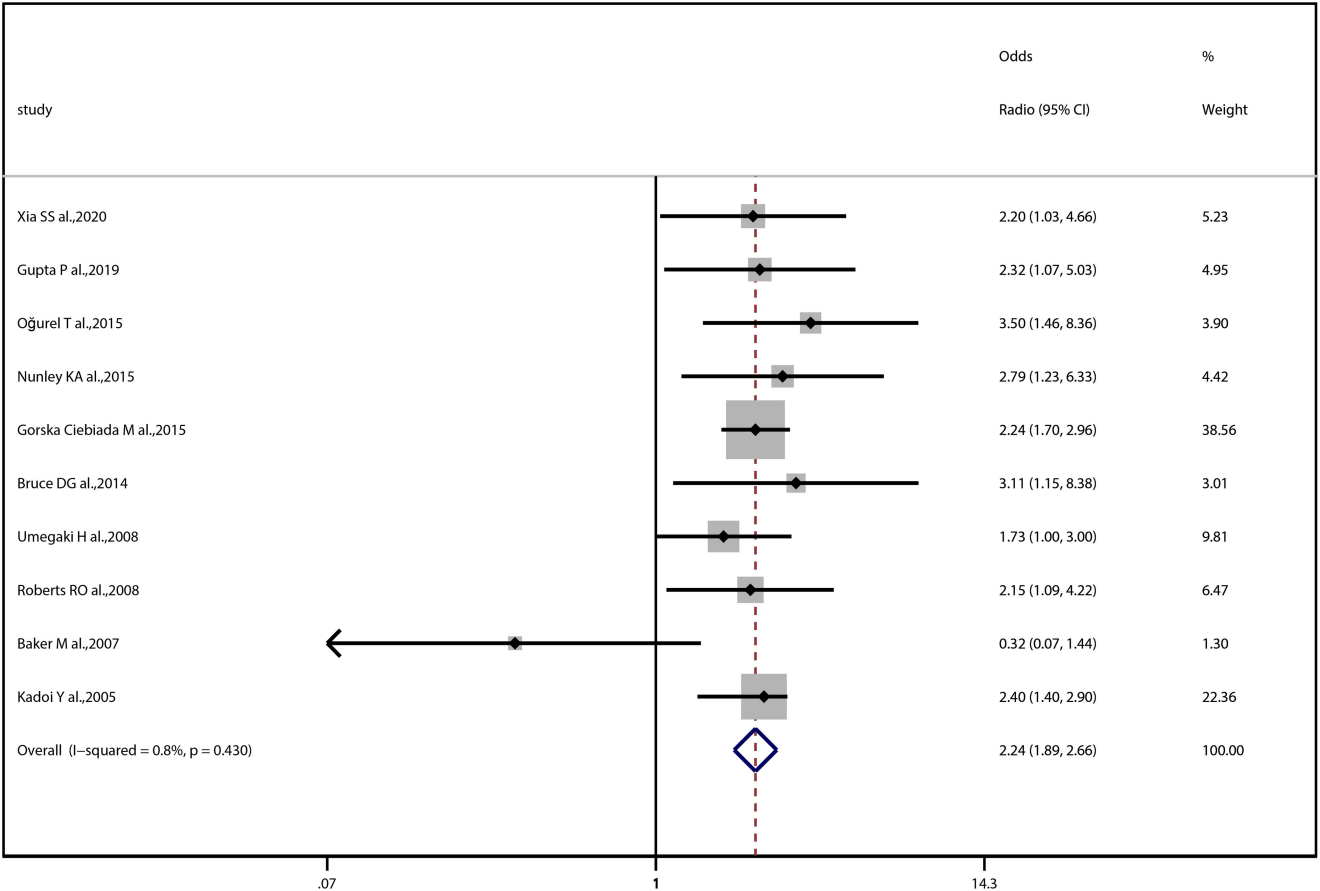
Forest plot and pooled estimates of the association between diabetic retinopathy and cognitive impairment. Each study corresponds to a horizontal line and a square. The size of the square represents the weight of the study in the pooled analysis, and the length of the horizontal line represents the 95% confidence interval (CI). The pooled fixed-effect estimate and its 95% CI are represented by a dashed vertical and a diamond. The vertical at 1 indicates that diabetic retinopathy is not associated with cognitive impairment.

**Figure 3:**
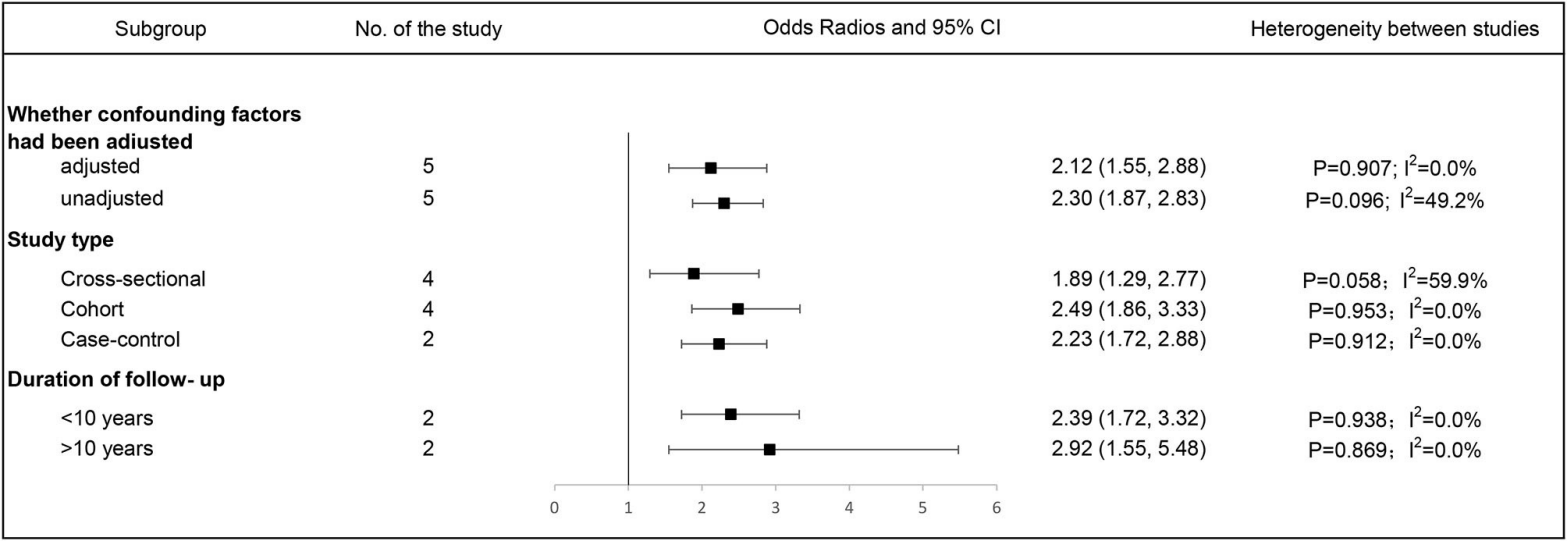
Subgroup analysis of the association between diabetic retinopathy and cognitive impairment. Forest plot and pooled estimates of the association between diabetic retinopathy and cognitive impairment, stratified by whether confounding factors had been adjusted, study type, and duration of follow-up. Each subgroup corresponds to a horizontal line and a square. The square represents the pooled estimate, and the length of the horizontal line represents the 95% confidence interval. The vertical at 1 indicates that diabetic retinopathy is not associated with cognitive impairment.

**Figure 4:**
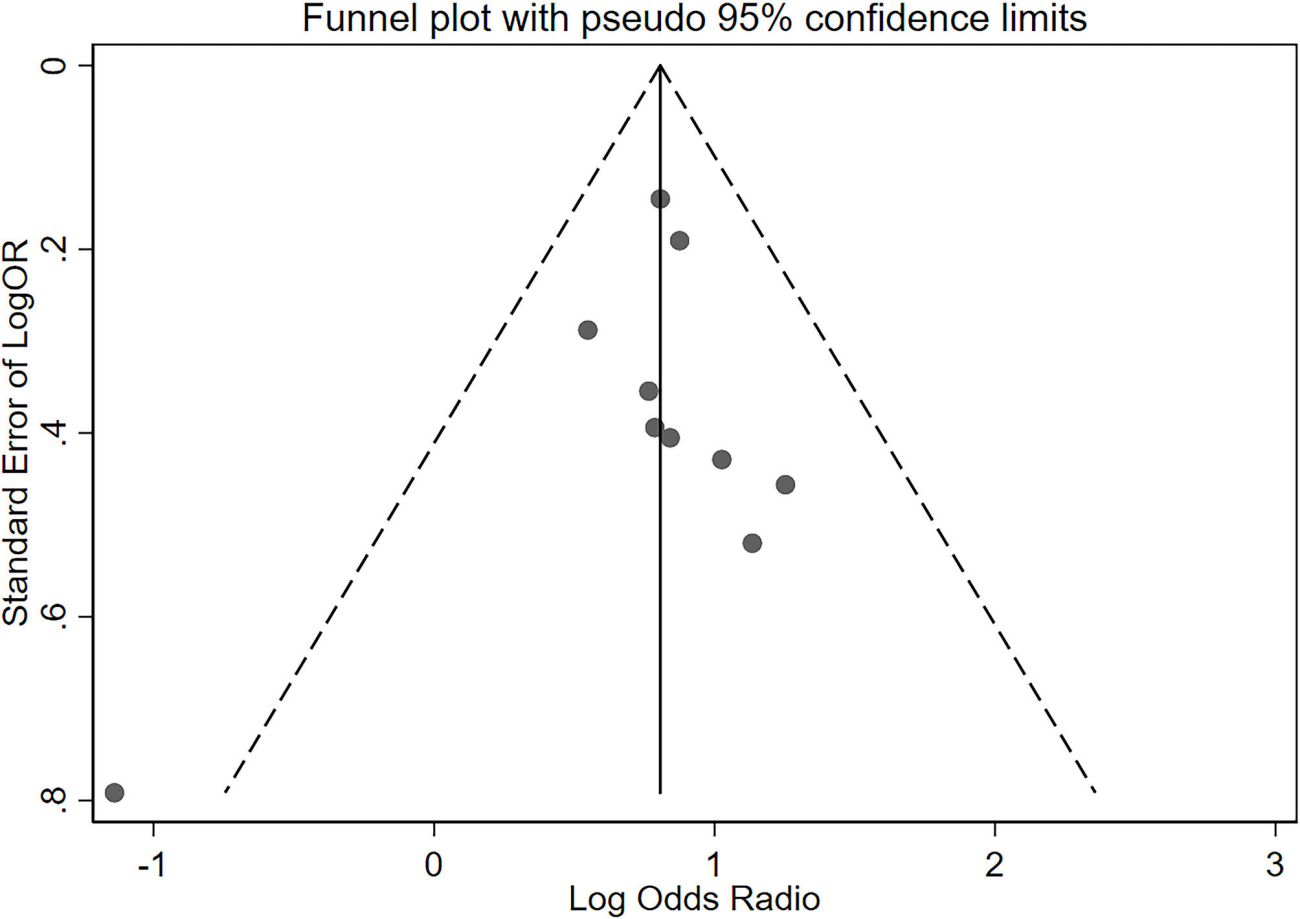
Funnel plot of standard error of log odds ratio (OR) for the association of diabetic retinopathy and cognitive impairment. The vertical line represents the summary estimate of log OR. Diagonal dashed lines estimate the expected distribution of studies; The Egger test did not show statistically significant asymmetry of the funnel plot (*P* = 0.538).

The OR of the association between DR and cognitive impairment was 2.24 (95% CI, 1.89–2.66; *I*^2^ = 0.8%), pooled from all included studies in Table 1 (Figure 2). The OR was 2.12 (95% CI, 1.55–2.88; *I*^2^ = 0.0%), pooled from studies in which confounding factors had been adjusted, and the OR was 2.30 (95% CI, 1.87–2.83; *I*^2^ = 49.2%), pooled from studies in which confounding factors had not been adjusted (Figure 3). When the comparison was stratified by study type, the association between DR and cognitive impairment was significant in all study types (Figure 3). When the comparison was stratified by the duration of follow-up, the association between DR and cognitive impairment was more significant with a follow-up of over 10 years (OR, 2.92; 95% CI, 1.55–5.48) than with a follow-up of less than 10 years (OR, 2.39; 95% CI, 1.72–3.32) (Figure 3). Finally, eliminating each of the included studies from the analysis did not affect the overall association between DR and cognitive impairment (Supplementary Figure 1). The Egger test did not show statistically significant asymmetry in the funnel plot (*P* = 0.538, Figure 4), indicating no significant publication bias.

The HRs of the association between DR and cognitive impairment were significant in four out of five studies, ranging from 1.09 to 1.32. Only one study showed that there was no connection between DR and cognitive impairment (HR, 1.12; 95% CI, 0.82–1.54).

### Grades of DR and Cognitive Impairment

**Figure 5:**
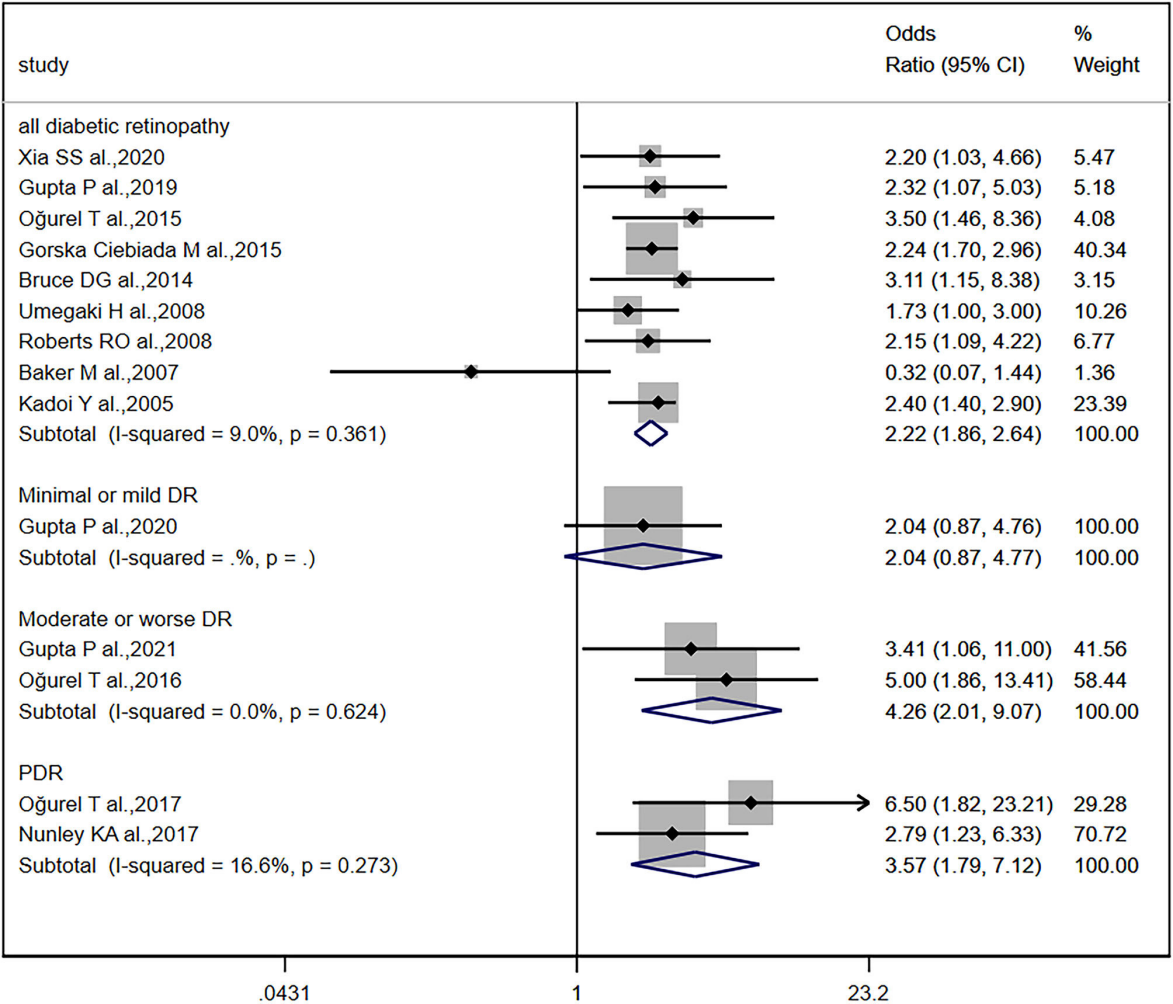
Grades of diabetic retinopathy and cognitive impairment. Forest plot and pooled estimates of the association between diabetic retinopathy and cognitive impairment, stratified by the grade of diabetic retinopathy. Each study corresponds to a horizontal line and a square. The size of the square represents the weight of the study in the pooled analysis, and the length of the horizontal line represents the 95% confidence interval (CI). The pooled fixed-effect estimate and its 95% CI are represented by a dashed vertical and diamond. The vertical at 1 indicates that diabetic retinopathy is not associated with cognitive impairment.

The distribution of studies by the estimate of the association between the grades of DR and cognitive impairment is plotted in Figure 5. Compared to other groups, minimal or mild DR was not significantly associated with cognitive impairment (OR, 2.04; 95% CI, 0.87–4.77). However, although proliferative diabetic retinopathy (PDR) was further distinguished, the association between PDR and cognitive impairment (OR, 3.57; 95% CI, 1.79–7.12; *I*^2^ = 16.6%) was not stronger than the association between moderate or worse DR and cognitive impairment (OR, 4.26; 95% CI, 2.01–9.07; *I*^2^ = 0.0%). Only one study reported HR data on patients with grades of DR, and a similar result was reported: compared to moderate or severe DR groups, mild DR was not significantly associated with cognitive impairment (HR, 1.5; 95% CI, 0.9–2.5).

## Discussion

In this systematic review and meta-analysis of observational studies, DR was found to be associated with cognitive impairment. Our meta-analysis provided evidence that patients with DR were more than twice as likely to develop cognitive impairment than were those without DR. The positive association was consistent across different study types, duration of follow-up, and regardless of whether confounding factors had been adjusted. In most of the included studies that reported HRs, there was also a positive association between DR and cognitive impairment. However, among the studies that provided HRs, the only study that found no link between DR and cognitive impairment was performed in a large group of patients with diabetes who survived to older ages (Rodill et al., 2018). As this study population included a healthy survivor group that outlived many peers, the relationship between DR and cognitive impairment might be underestimated.

Although DR is a common microvascular complication of diabetes, only one systematic review has previously investigated its association with cognitive impairment (Crosby-Nwaobi et al., 2012). However, only three studies were included in the systematic review, and the only cohort study was not population-based. In addition, the cohort study in that systematic review utilized a group of people who were about to undergo coronary artery bypass grafting, which might cause a certain degree of bias and reduce the applicability of the results. Moreover, the previous systematic review did not show whether patients with moderate to worse DR had more severe cognitive impairment than those without or with mild DR. Therefore, the current study attempted to quantitatively analyze the relationship between diabetic retinopathy and cognitive impairment based on more studies, especially cohort studies, and further explore the relationship between the degree of DR and cognitive impairment.

We found that minimal or mild DR was not significantly associated with cognitive impairment, but moderate to worse DR or PDR was strongly associated with cognitive impairment. Crosby-Nwaobi et al. found that patients with no or minimal DR demonstrated more cognitive impairment than did those with PDR (Crosby-Nwaobi et al., 2013), and this conclusion was inconsistent with ours. However, there were two important limitations in the previous study that may account for the discrepancy between their conclusions and ours. First, the authors combined people without DR with mild DR as a control group, which may have weakened the association between PDR and cognitive impairment. Second, the level of education was different between the no/mild DR and PDR groups. Education level is associated with cognitive impairment, which may have influenced the final results.

Unfortunately, the mechanisms underlying the association between DR and cognitive impairment have not been well-explained. DR highlights hyperglycemia-induced microvascular damage as a specific complication of diabetes. The relationship between hyperglycemia and microvascular dysfunction is bidirectional and constitutes a vicious cycle (Stehouwer, 2018). Due to retinal vascular shared origin and drainage with the cerebrovascular circulation (Moss, 2015), one possible hypothesis is that DR may indicate diabetes-induced microvascular changes in the brain, which may ultimately cause cognitive impairment. Neurovascular coupling dysfunction and destruction of the blood–brain barrier are common in diabetic cerebrovascular dysfunction (van Sloten et al., 2020). Similarly, neurovascular coupling dysfunction (Garhöfer et al., 2020) and destruction of the blood–retinal barrier (Starr et al., 2003) are common in DR. This suggests that DR and cerebral microangiopathy have similar pathophysiological changes. This idea is further supported by the presence of microbleeds (Woerdeman et al., 2014), white matter lesions, and lacunes (Sanahuja et al., 2016) in the brains of patients with DR. Diabetes cerebral microvascular dysfunction and damage may lead to ischemia, hemorrhage, abnormal neuronal function, neuronal cell death, and altered neuronal connectivity, which contribute to cognitive dysfunction (van Sloten et al., 2020). The proximity of the onset of retinopathy to the onset of some cognitive domain damage also seems to support this idea. Within 5 years of diagnosis, 14% of patients with type 1 diabetes and 33% of patients with type 2 diabetes may develop DR (Cheung et al., 2010), and verbal memory and fluency are also likely to decline (Callisaya et al., 2019).

However, this hypothesis about the mechanism of the association between DR and cognitive impairment cannot fully explain the results of the current meta-analysis and systematic review. First, if this hypothesis is correct, then since the severity of DR is associated with the risk of cerebral microangiopathy (Modjtahedi et al., 2020), the severity of DR should also be associated with the risk of cognitive impairment. However, what was puzzling was that although some included studies focused on the relationship between PDR and cognitive impairment (OR, 3.57; 95% CI, 1.79–7.12; *I*^2^ = 16.6%), the relationship was not stronger than that between moderate to worse DR and cognitive impairment (OR, 4.26; 95% CI, 2.01–9.07; *I*^2^ = 0.0%). A possible explanation could be that moderate to worse DR included PDR in most of the included studies, and, therefore, its association with cognitive impairment has been overestimated. Second, in most of the cohort studies we included, why was there still a time gap between the onset of diabetic retinopathy and cognitive impairment, and the longer the time gap, the stronger the relationship? We speculated that it may be because even though cerebrovascular and some cognitive domains are damaged early, it still needs a certain degree of time to accumulate before it can be reflected in global cognitive tools, but this speculation still lacks necessary evidence. Third, the tools used to evaluate cognitive impairment, such as the Mini-Mental State Examination and Montreal Cognitive Assessment, can only evaluate the cognitive level of patients but cannot confirm the occurrence of cerebral microvascular disease. Therefore, the results of this meta-analysis and systematic review do not provide strong support for this hypothesis. More evidence is needed to confirm whether there is a clear mechanistic link between DR and cognitive impairment.

The included cohort studies that reported HRs mostly showed that DR could predict dementia (Table 2). Dementia typically occurs after the age of 65–70 years, and no evidence exists that diabetes increases the risk of early-onset dementia (Biessels et al., 2014). This may indicate that different stages of cognitive impairment in patients with diabetes should not be regarded as a continuum (Biessels and Despa, 2018). Our results suggest that strategies that focus on DR screening may be useful in identifying individuals at risk of dementia, which could expand the role of diabetic retinopathy screening.

## Limitation

There are some limitations to this systematic review and meta-analysis. First, whether PDR, the most severe form of DR, is most strongly associated with cognitive impairment remains unresolved. If the risk of cognitive impairment is related to the grade of retinopathy, the mechanistic link between DR and cognitive impairment is more plausible. However, although researchers were aware of the problem, PDR was not separated from moderate or worse DR. Second, some studies have reported that mild DR was not associated with cognitive impairment, but we were not able to obtain the data. Third, because some studies did not report adjustments or reported incomplete adjustments for potential confounders, we were not able to combine models with studies that adjusted for the same set of confounding factors. Fourth, because the definitions of DR and cognitive impairment varied in the included studies, we did not compare the results for different retinopathy diagnostic criteria, cognitive impairment diagnostic criteria, or cognitive testing tools. Fifth, since the included studies evaluated global cognitive function, we were unable to assess the relationship between DR and different cognitive domains. Sixth, many of the included studies did not specify the type of diabetes, even though the mechanisms of cognitive impairment caused by different types of diabetes may differ (McCrimmon et al., 2012). Finally, there is clear evidence of sex differences in cognitive impairment, and the rate of cognitive decline with aging is also different between the sexes (Li and Singh, 2014). However, because most of the included studies adjusted for sex as a confounding factor and the lack of information on retinopathy in sex, we were unable to use these studies to analyze males and females separately. A previous study suggested that negative associations between DR and several cognitive measures were statistically significant only in males (Ding et al., 2010), but the significantly greater number of men with DR than females in this study may have exaggerated the results. Therefore, further research is needed to confirm whether the relationship between DR and cognitive impairment differs between the sexes.

## Conclusion

In conclusion, the present systematic review and meta-analysis demonstrated that DR is associated with an increased risk of cognitive impairment. Screening for DR may help identify individuals with cognitive impairment at an earlier stage. More studies are needed to confirm the association between PDR and cognitive impairment.

## Data Availability Statement

The original contributions presented in the study are included in the article/Supplementary Material, further inquiries can be directed to the corresponding authors.

## Author Contributions

GW and GN contributed to conception and design of the study. DC, XZ, and SY organized the database. DC performed the statistical analysis and wrote the first draft of the manuscript. All authors contributed to manuscript revision, read, and approved the submitted version.

## Conflict of Interest

The authors declare that the research was conducted in the absence of any commercial or financial relationships that could be construed as a potential conflict of interest.
