# Supplementary material for: Association Between Diabetic Retinopathy and Cognitive Impairment: A Systematic Review and Meta-Analysis
Source: Front Aging Neurosci. 2021 Jun 30;13:692911. doi: 10.3389/fnagi.2021.692911 (PMC8278198; doi:10.3389/fnagi.2021.692911)
Supplement: Supplementary file 1 [file Table_1.docx]

**Supplementary Table 1 The quality of cross-sectional studies**

| **Item** | Xia et al. (2020) | Oğurel et al.  (2015) | Umegaki et al. (2008) | Baker et al. (2007) |
| --- | --- | --- | --- | --- |
| 1) Define the source of information (survey, record review) | Yes | Yes | Yes | Yes |
| 2) List inclusion and exclusion criteria for exposed and unexposed subjects (cases and controls) or refer to previous publications | No | Yes | Yes | Yes |
| 3) Indicate time period used for identifying patients | Yes | Yes | Yes | Yes |
| 4) Indicate whether or not subjects were consecutive if not population-based | Unclear | Yes | Unclear | Yes |
| 5) Indicate if evaluators of subjective components of study were masked to other aspects of the status of the participant | Unclear | Unclear | Unclear | Unclear |
| 6) Describe any assessments undertaken for quality assurance purposes (e.g., test/retest of primary outcome measurements) | Yes | Yes | Yes | Yes |
| 7) Explain any patient exclusions from analysis | Yes | No | Yes | Yes |
| 8) Describe how confounding was assessed and/or controlled. | Yes | No | Yes | Unclear |
| 9) If applicable, explain how missing data were handled in the analysis | Unclear | Unclear | Unclear | Unclear |
| 10) Summarize patient response rates and completeness of data collection | Unclear | Unclear | Unclear | Unclear |
| 11) Clarify what follow-up, if any, was expected and the percentage of patients for which incomplete data or follow-up was obtained | Unclear | Unclear | Unclear | Unclear |
| [Quality](javascript:;) [assessment](javascript:;) | moderate | moderate | moderate | moderate |
