# Supplementary material for: Association Between Diabetic Retinopathy and Cognitive Impairment: A Systematic Review and Meta-Analysis
Source: Front Aging Neurosci. 2021 Jun 30;13:692911. doi: 10.3389/fnagi.2021.692911 (PMC8278198; doi:10.3389/fnagi.2021.692911)
Supplement: Supplementary file 2 [file Table_2.docx]

**Supplementary Table 2 The quality of cohort studies or case-control studies**

| **Study** | Selection | Comparability | Exposure/Outcome | [Quality](javascript:;) [assessment](javascript:;) |
| --- | --- | --- | --- | --- |
| Cohort | | | | |
| Yu et al.  (2020) | ★★★★ | ★ | ★ | moderate |
| Gupta et al.  (2019) | ★★★★ | ★★ | ★ | high |
| Deal et al.  (2019) | ★★★★ | ★★ | ★★★ | high |
| Lee et al.  (2019) | ★★★★ | ★★ | ★★ | high |
| Rodill et al.  (2018) | ★★★★ | ★ | ★★ | high |
| Nunley et al.  (2015) | ★★★ | ★ | ★★ | moderate |
| Bruce et al.  (2014) | ★★★ | - | ★★ | moderate |
| Exalto et al.  (2014) | ★★★★ | ★★ | ★★ | high |
| Kadoi et al.  (2005) | ★★★ | - | ★★ | moderate |
| Case-control | | | | |
| Gorska-Ciebiada et al.  (2015) | ★★★★ | - | ★ | moderate |
| Roberts et al.  (2008) | ★★★★ | ★★ | ★ | high |
