# Supplementary material for: Association Between Diabetic Retinopathy and Cognitive Impairment: A Systematic Review and Meta-Analysis
Source: Front Aging Neurosci. 2021 Jun 30;13:692911. doi: 10.3389/fnagi.2021.692911 (PMC8278198; doi:10.3389/fnagi.2021.692911)
Supplement: Supplementary file 3 [file Data_Sheet_1.PDF]

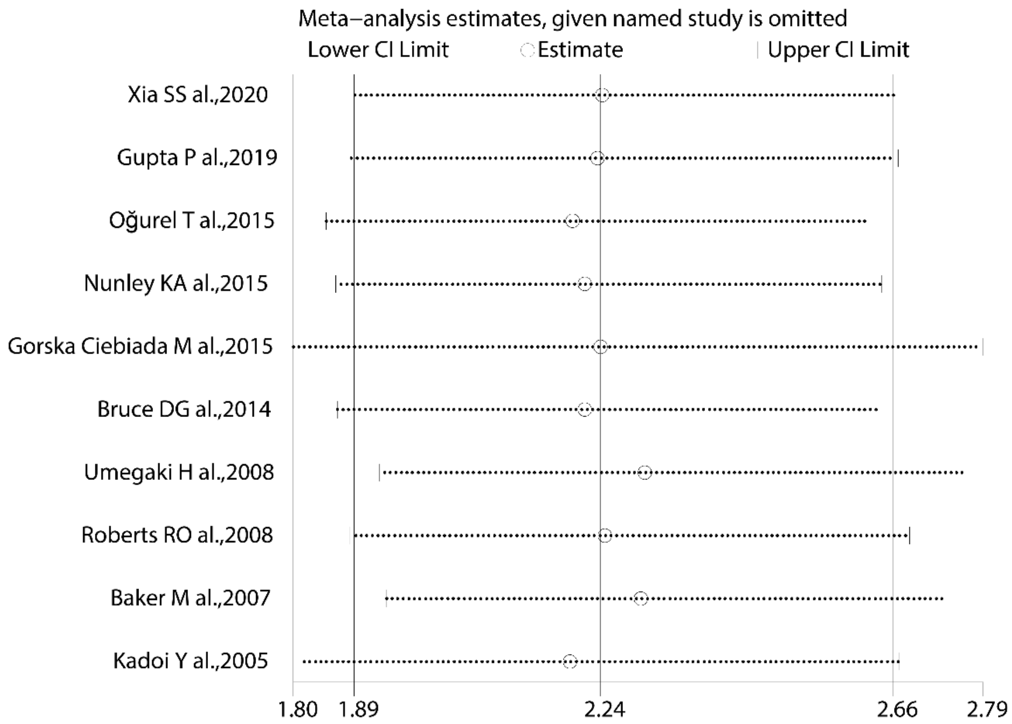

Supplementary Figure 1 Sensitive analysis of the association of diabetic retinopathy and cognitive impairment. Each study corresponds to a horizontal line and a circle. The circle represents the pooled estimate of the other studies after omitting the given named study, and the length of the horizontal line represents the 95% confidence interval.
